# Requirements and Design of the PROSPER Protocol for Implementation of Information Infrastructures Supporting Pandemic Response: A Nominal Group Study

**DOI:** 10.1371/journal.pone.0017941

**Published:** 2011-03-28

**Authors:** Toomas Timpka, Henrik Eriksson, Elin A. Gursky, Magnus Strömgren, Einar Holm, Joakim Ekberg, Olle Eriksson, Anders Grimvall, Lars Valter, James M. Nyce

**Affiliations:** 1 Department of Medical and Health Sciences, Linköpings universitet, Linköping, Sweden; 2 Department of Computer Science, Linköpings universitet, Linköping, Sweden; 3 National Strategies Support Directorate, ANSER/Analytic Services Inc, Arlington, Virginia, United States of America; 4 Department of Social and Economic Geography, Umeå University, Umeå, Sweden; 5 Department of Anthropology, Ball State University, Muncie, Indiana, United States of America; Massey University, New Zealand

## Abstract

**Background:**

Advanced technical systems and analytic methods promise to provide policy makers with information to help them recognize the consequences of alternative courses of action during pandemics. Evaluations still show that response programs are insufficiently supported by information systems. This paper sets out to derive a protocol for implementation of integrated information infrastructures supporting regional and local pandemic response programs at the stage(s) when the outbreak no longer can be contained at its source.

**Methods:**

Nominal group methods for reaching consensus on complex problems were used to transform requirements data obtained from international experts into an implementation protocol. The analysis was performed in a cyclical process in which the experts first individually provided input to working documents and then discussed them in conferences calls. Argument-based representation in design patterns was used to define the protocol at technical, system, and pandemic evidence levels.

**Results:**

The Protocol for a Standardized information infrastructure for Pandemic and Emerging infectious disease Response (PROSPER) outlines the implementation of information infrastructure aligned with pandemic response programs. The protocol covers analyses of the community at risk, the response processes, and response impacts. For each of these, the protocol outlines the implementation of a supporting information infrastructure in hierarchical patterns ranging from technical components and system functions to pandemic evidence production.

**Conclusions:**

The PROSPER protocol provides guidelines for implementation of an information infrastructure for pandemic response programs both in settings where sophisticated health information systems already are used and in developing communities where there is limited access to financial and technical resources. The protocol is based on a generic health service model and its functions are adjusted for community-level analyses of outbreak detection and progress, and response program effectiveness. Scientifically grounded reporting principles need to be established for interpretation of information derived from outbreak detection algorithms and predictive modeling.

## Introduction

A recent evaluation of national pandemic response polices found that the regional and local information infrastructures for collecting and processing pandemic data are not aligned with response program processes and structures [1]. The infrastructures needed differ in several aspects from traditional health information systems because they are to be used in situations when outbreaks threaten to overwhelm first-order information resources nationally and locally on hand for infectious disease control [2], [3]. This occurred in 2009 with the emergence of a novel A (H1N1) influenza virus (the ‘swine flu’) in Mexico. If the resources in the information infrastructure used in such exceptional situations are poorly validated and coordinated, then the information that is produced may delay or even mislead response program implementation [4], [5]. For instance, when the 2009 influenza outbreak had progressed beyond pandemic levels 2 and 3, public health officials in a rapidly increasing number of nations had to make decisions about appropriate response actions. In the absence of a vaccine, the closure of schools with infected pupils was used by some countries, but not others. In the USA, the CDC initially supported school closures, while the Public Health Agency of Canada did not recommend this action. The UK Health Protection Agency took the position that “consideration should be given to temporarily closing a school” [6]. In Sweden, a decision was made to immunize the entire population. Similar decisions were made in the UK, France, Ireland, Finland and Greece, while most other European countries chose other vaccination strategies. Hence, even though their action thresholds may have differed for various reasons, it is unmistakable that policy-makers in comparable countries arrived at different decisions concerning pandemic response strategies. In other words, despite the availability of advanced information systems for early laboratory diagnosis and communication of virological data during the initial phases of the outbreak, the planning of further action did not appear to have been derived from shared evidence. One reason for this may be that there was no integrated information infrastructure in place that adequately could support coordinated planning of regional and local pandemic response during the later stages of the outbreak.

This paper sets out to derive a protocol that can be used to implement an information infrastructure supporting pandemic response programs at the stages when containment of the outbreak at its source is no longer possible, i.e. for the support of national and local responses in the organizational context(s) where the corresponding public health agencies operate [7]. At these stages (at pandemic levels 4 to 6), the microbiological characteristics of the infectious agent can be expected to have been established [8].

## Methods

### Data collection

The nominal group technique [9] was used to collect and analyze requirements data. This technique is a semi-formal decision-making method for groups. Every member of the group gives their view of the solution, with a short explanation. Then, duplicate solutions are eliminated from the list of all solutions, and the members proceed to rank the solutions. A facilitator encourages the sharing and discussion of reasons for the choices made by each group member, thereby identifying common ground, and a plurality of ideas and approaches. This diversity may allow the creation of a hybrid idea, combining parts of two or more ideas. In the basic method, the numbers each solution receives are totaled, and the solution with the most favored ranking is selected as the final decision. In this study, a review of the literature on pandemic information management practices was first performed. Two expert panels were thereafter formed to outline requirements on data sources and analytic functions for an information infrastructure for pandemic response programs. The nominal group technique was used to identify strengths versus areas in need of development, as well as used as a decision-making voting alternative. Options were not always numerically ranked, but also evaluated more subjectively. Individual experts reviewed a working requirements document followed by telephone conference discussions. Requirements on the data sources were defined by a panel consisting of scientists and practitioners (n = 8) with backgrounds in medicine, epidemiology, medical anthropology, statistics, computer science, health informatics, and socio-economic geography. The panel examining requirements on analytic functions consisted of scientists and practitioners (n = 5) with backgrounds in medicine, statistics, computer science, health informatics and cognitive science. The experts provided a first round of comments to a requirements process coordinator, who assembled these into requirements specification documents. The group analyzing the data sources produced an overview of the status of the present infrastructure available for national and local response programs. They thereafter concentrated on practical issues related to pandemic planning. For example, the group reviewed outbreak-related data and their sources, and the literature concerning the social behavior during an ongoing pandemic. The functions group identified requirements on outbreak detection and forecasting methods. When subsequent turns did not return significant changes in the documents, the requirement specifications were considered to be established.

### Data analysis

A formal argument-based method for reaching consensus on complex problems [10], [11] was used for analyzing requirements data. Here, members of the two panels were merged into one protocol specification group. The task communicated to the group was to formulate a protocol design using the requirements, their expertise, and the published literature. The experts first provided their individual comments, which were collected by a design process coordinator. Functional protocol solutions were formulated independently by experts who reviewed a document that outlined design patterns describing the protocol. Inter-connected design patterns were used because they can communicate the functionality of a design in a way that is understandable to a variety of non-expert stakeholders [12]. Each pattern language represents a specific problem and describes a possible solution. In the present analysis, the design patterns were represented in the form Title, Problem-Requirements, Design, and Examples. The Examples section provides illustrations of how a design can be implemented in order to address a particular information problem in pandemic response. Comments on subsequent versions of the design patterns were circulated to the entire expert group, and a consensus document was established describing a final set of design patterns. In the third and final step, the design patterns were summarized into a final protocol. After having formulated the protocol, each expert panel member was asked to report possible disagreements with the protocol (Text S1).

## Results

### Protocol requirements

The review of the literature on pandemic information management practices showed that major present obstacles were a shortage of reliable data on populations' disease and susceptibility status and a lack of validated outbreak detection and forecasting methods (Text S2). The requirements on the data to be handled in an information infrastructure supporting pandemic response were subdivided into specifications of socio-immuno-geographic data to be collected to describe communities, the quality and timeliness of epidemiological outbreak data, and on how data and assumptions about population behavior are managed (Text S3). The most important requirement on analytic functions to be supplied by the infrastructure was that the functions could work in routine surveillance and monitoring of intervention effectiveness in public health practice, and not just in temporary trials of response program components (Text S4).

### Protocol design

**Figure 1:**
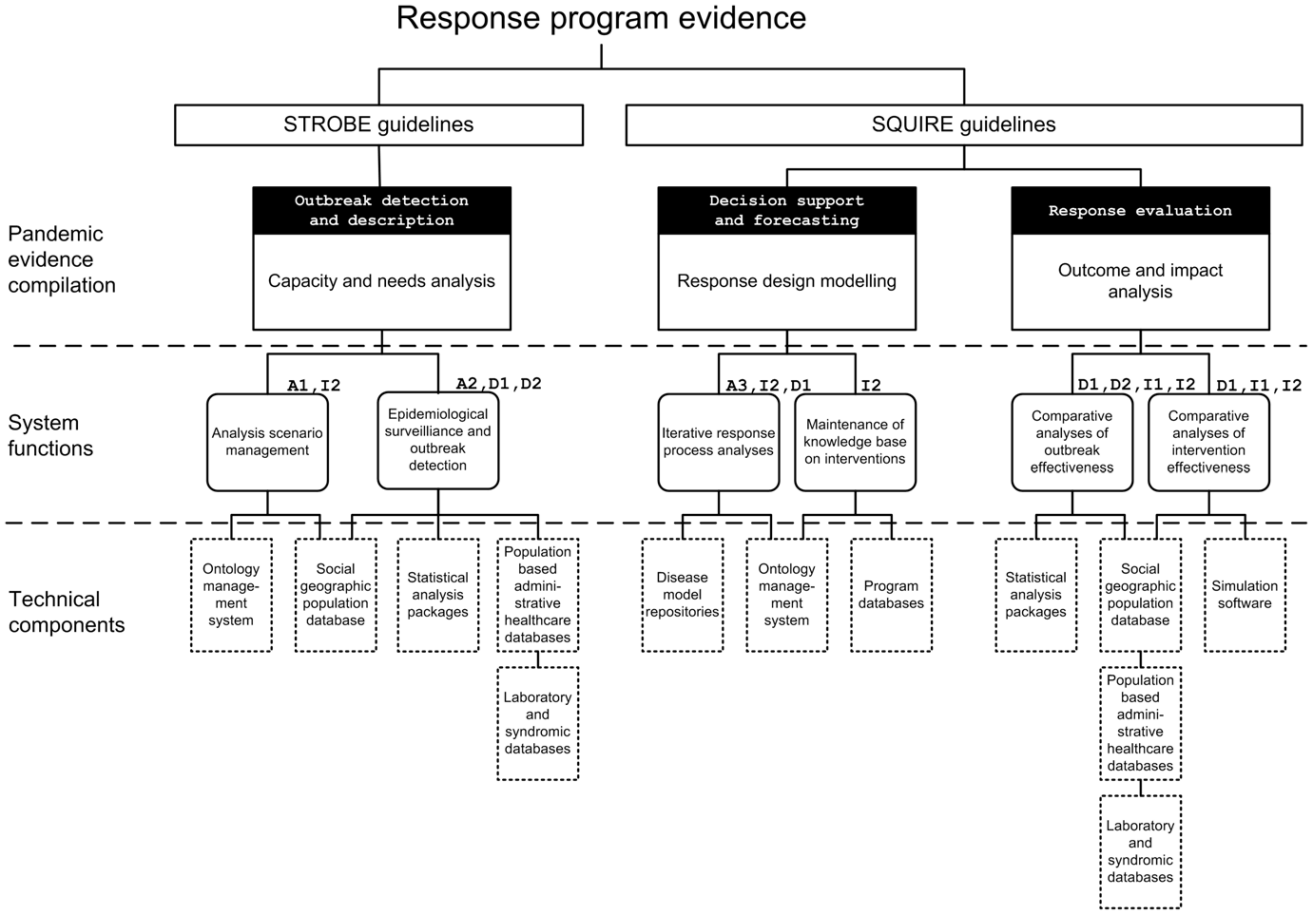
The PROSPER protocol for implementation of a standardized information infrastructure for evidence-based pandemic response. Cross-references are provided in the protocol to design patterns at evidence and functional levels.

**Figure 2:**
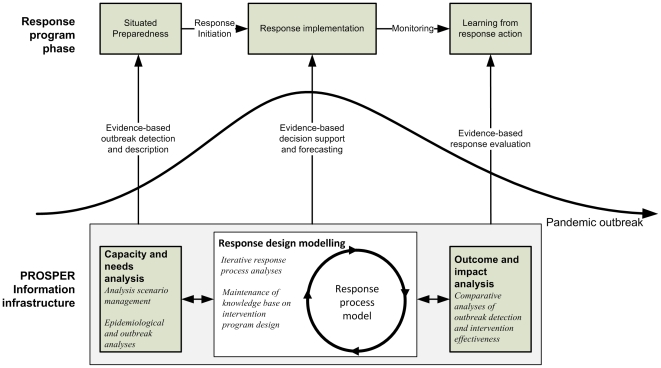
Display of the PROSPER protocol in relation to infectious disease response program implementation. During inter-pandemic phases, Capacity and needs analysis (CNA) functions are used for examination and surveillance of the community. In support of early situated preparedness, the impact from alternative response measures, such as social distancing, are already in this phase preliminarily estimated using community data supplied by the CNA functions and forecasting methods available through Response design modeling (RDM) functions. When a pandemic alert is issued by the WHO, the outbreak detection algorithms included in the CNA functions are calibrated with regard to the most recent information about the infectious agent and local circumstances. During the early stages of a detected local outbreak, also the parameters used by the RDM functions are adjusted as more information on transmission characteristics and genomic features of the agent becomes available. When policy-makers prepare to decide about first local response measures, such as closing schools or issues antiviral prophylaxis schemes, results from the progressing analyses of the local community are used and compared with recommendations from the WHO and other external sources. If an early response measure is decided, it can be monitored through the RDM functions and data from the CNA functions. When the monitoring indicates that the outbreak is decreasing in strength, the local policy-makers can use this information in lieu with reports of the global progress of the pandemic to withdraw restricting measures, e.g. social distancing. Following the outbreak, the precision of the forecasts and effectiveness of the interventions can be analyzed using the Outcome and impact assessment (OIA) functions.

The Protocol for a Standardized information infrastructure for Pandemic and Emerging infectious disease Response (PROSPER) outlines an information infrastructure for pandemic response that is aligned with regional and local response programs. The infrastructure covers information resources for community surveillance and initiation of response, iterative design of response processes, and examination of outcomes and impacts. For each of these areas, PROSPER describes a supporting information infrastructure in three hierarchically related levels, from technical components and system functions to pandemic evidence compilation (Figure 1). The technical components can be compiled using conventional information system methods by regional and local public health agencies or by other organizations tasked with responding to a pandemic threat. An example of how an information infrastructure based on PROSPER can support planning, performance, and evaluation of local and regional response during a pandemic outbreak is provided in Figure 2.

The system functions for capacity and needs analysis (CNA), response design modeling (RDM), and outcome and impact analysis (OIA) in PROSPER reflect the methods used to produce pandemic evidence and the organization of infectious disease response. The implementation of these functions is described at the technical component level using examples.

### Capacity and needs analysis

The CNA functions are supplied by computer hardware and software for scenario management and data access for epidemiological surveillance and outbreak detection. The Strengthening the Reporting of Observational Studies in Epidemiology (STROBE) recommendations [13] specifying what should be included in accurate reports of observational studies are used to organize the communication of results from the analyses at the evidence level.

### CNA1 Analysis scenario management

#### Problem-Requirements

A1 Socio-immuno-geographical representation of communities. I2 Explicit fact and hypothesis management.

#### Design

Spatially explicit representations of communities are used to allow experiments with factual and synthetic populations. This design solution allows different scenarios to be defined by changing the socio-immuno-geographical starting conditions of the analysis model. Basic model categories included in the pandemic outbreak scenario define homes, transportation systems, and other geographic conditions, e.g. location of workplaces, schools, shopping malls, and facilities for sports and entertainment events. Besides personal variables, such as immunological status, the representations also include relational variables, such as individual-mother, -partner, -child, and -co-worker at workplace. These relational variables allow for modeling and representing a substantial part of the social networks that transmit infectious agents. In other words, socio-geographical preprocessing of spatially explicit population data can be used to, in advance, identify specific groups and populations that may require more careful and intensified surveillance.

#### Examples

The scenario management can be based on a ontology handling system [14] and computer-based models for socio-immuno-geographical representation of populations [15]. Settings for increasingly detailed scenario models can be developed, representing, e.g. local social interaction and commuting patterns [16].

### CNA2 Epidemiological surveillance and outbreak detection

#### Problem-Requirements

A2 Control and visualization of data quality and timeliness. D1 Access and adjustments to data. D2 Integration of multiple context-specific detection algorithms.

#### Design

Epidemiological data from actual outbreaks are collected and stored in networked databases and complemented with artificially generated data. This integration of data supports both detailed analyses of ongoing outbreaks and experiments on hypothetical outbreaks in populations. The factual outbreak data range from highly specific genomic and microbiological laboratory data [17], [18] to non-specific syndromic data, e.g. from telephone health advice centres and Internet website logs. It is strongly recommended that data sources are controlled by methods that allow for systematic statistical follow-up of the data used. In particular, this approach can address short-term trends in the pandemic progress that are easily masked by errors in sampling or laboratory practices. Statistical tools for trend analysis, such as semi-parametric regression models [19], are used to identify causes of flaws in data collection routines that can lead to erroneous interpretations. Interactive graphs (http://www.ggobi.org) and motion chart (http://www.gapminder.org) services available on the Internet are used for obtaining overviews of large data sets. Studies have shown that epidemiologists using human visual pattern-recognition capacities can signal epidemiological alerts from “image walls” presenting local, regional and/or national surveillance patterns even though the patterns passed unnoticed through conventional systems [20]. The design is based on outbreak detection algorithms that are context sensitive. The performance and timeliness of spatial, temporal and spatio-temporal algorithms can be connected to particular settings. Global sensitivity and uncertainty analyses are supported in order to take into account the features of separate sets of data sources, outbreak detection algorithms, and the interaction between these in an integrated system [21], [22].

#### Examples

With the recent observation of new highly pathogenic H5N1 and H7N7 strains, and the appearance of the influenza pandemic caused by the H1N1 swine-like lineage, collaborative efforts to share observations on the influenza virus in both animals and humans has been established. Open access genomic databases are available over the Internet, which facilitates the identification of locally and regionally circulating viruses. The OpenFlu database (OpenFluDB; http://openflu.vital-it.ch) [23] contains genomic and protein sequences, as well as epidemiological data from more than 27,000 isolates. The isolate annotations include virus type, host, geographical location and experimentally tested antiviral resistance. Administrative healthcare databases [24] can be used to assemble geographically explicit case data at multiple levels. In addition to tabulations, these data can also be visualized graphically and by using motion charts (http://www.crisim.org). Influenza diagnoses recorded at primary care centers can hereby be used to track the disease progress in the community, while data from hospital wards and intensive care units can be used to establish the proportion of severe cases in different population strata. Moreover, telenursing services are in many countries supported by telehealth Electronic Patient Records (tEPRs), where the reason for contact and the residence of each caller is documented [25], [26]. Databases that collect data from regional tEPRs can be used for surveillance and early detection of infectious disease outbreaks. Other sources of syndromic data available in many communities include school absence records and software monitoring visits at public health websites. Outbreak detection can be performed in the administrative healthcare database environment [24]. The relevant algorithms can be integrated with the database managements systems to facilitate ease of use. Detection methods with specific characteristics advantageous for influenza outbreak detection in such databases can be developed. Alarm levels can here be set with regard to the sensitivity and specificity that is suitable for the particular community context at hand.

### Response design modeling

The RDM design patterns outline how the corresponding functions are supplied by hardware and software for response process analysis and knowledge-base maintenance. The Standards for Quality Improvement Reporting Excellence (SQUIRE) guidelines for reporting studies of quality improvement in health services [27] are used to organize the results of these analyses at the evidence level.

### RDM1 Iterative response process analyses

#### Problem-Requirements

A3 Explicit representation of populations over time. D1 Access and adjustments to data. I2 Explicit fact and hypothesis management.

#### Design

Analyses of outbreak response program components using simulated interventions and historical or virtual data are employed until real-time surveillance data become available and evaluations of factual interventions are feasible. The early disease models used in the virtual analyses are derived from the literature, e.g. with regard to incubation period and serial interval. Response program components are specified as intervention models. Public health analysts can prepare analyses of response processes by configuring program components and specifying intervention model parameters, e.g. the prophylactic performance of specific antiviral drugs or drug combinations.

#### Examples

In the simulation environment, the software for the management of the response program models and the software for the execution of the analyses are preferably separated [28]. Such separation allows for flexible modeling of unexpected events and circumstances, while maintaining the run-time performance of simulation programs. Disease and intervention characteristics are available from profiles reported in the literature [29] and on the Internet (https://www.epimodels.org/midas/modelProfilesFull.do). These characteristics can be combined to obtain a typology of basic models and baseline parameter settings.

### RDM2 Maintenance of a knowledge base on interventions

#### Problem-Requirements

D1 Access and adjustments to data. I2 Explicit fact and hypothesis management.

#### Design

To support an iterative response program design, each program configuration is stored together with the corresponding simulation results in a program database. This makes it possible to track and report algorithms and the effectiveness of different response program components under particular preconditions. For each simulation cycle, the models used in program representation, parameter settings (literature-derived and assumption-based), and data sources are documented together with outcomes.

#### Examples

An ontology handling system can act both as a model configuration manager and as a model archive. Components of previously analyzed interventions can be stored as library items. Separate interventions can be combined into multi-component intervention programs, and their collective effectiveness rapidly estimated by simulations. The assumptions used for the analyses can be made explicit by an assumptions tracing function for each specific class of analyses [30], and a report function can be used to compile displays that specify the assumptions underlying each evaluation result [31].

### Outcome and impact analysis

The OIA section of the protocol provides an outline for analyses of outbreak detection and intervention effectiveness. The SQUIRE guidelines for reporting studies of quality improvement in health services [27] are used when communicating results from the analyses at the evidence level. The functions in this section are based on computer hardware and software that normally not are used by regional and local public health departments. However, these resources can today be accessed or acquired without major financial investments by utilizing open source software and short-term rental of computing power via the Internet, e.g. through Amazon's Elastic Computing Cloud (EC2).

### OIA1 Comparative analyses of outbreak detection effectiveness

#### Problem-Requirements

D1 Access and adjustments to data. D2 Integration of multiple context-specific detection algorithms. I1 Comparative studies of intervention strategies. I2 Explicit fact and hypothesis management.

#### Design

Evaluations are focused on comparisons between different outbreak detection methods and their components in specified socio-geographical environmental settings and real-world contexts.

#### Examples

Comparative assessments can be performed using both databases containing data from historical and current influenza outbreaks as well as from synthetic datasets. The major part of the assessments can be performed in the administrative healthcare database environment [24]. Detection methods with specific characteristics advantageous for influenza outbreak detection in such databases can thereby be developed. Comparative analyses can be performed using the CUSUM methods [32] and SatScan software [33] as references.

### OIA2 Comparative analyses of response intervention effectiveness

#### Problem-Requirements

D1 Access and adjustments to data. I1 Comparative studies of intervention strategies. I2 Explicit fact and hypothesis management.

#### Design

Two types of analyses are supported: forecasting comparisons of different intervention alternatives before or during an outbreak, and comparisons between forecasted and actual outcomes. Because forecasts are highly context-dependent, the design focuses on analyses of intervention effectiveness. Comparative forecasts of intervention effectiveness are typically based on differences in outcome as measured by, e.g. disease reproduction rates, epidemic curves with daily new cases (attack rates per geographic region), and burden of illness in different vocational groups. Assumptions used are explicitly specified in the definition of program components and disease models. For example, if there is a lack of information on local school structures, it is possible to document that the administrative organization of local elementary schools used in a community model is an assumption rather than a verified fact. In comparative analyses performed after an outbreak, the forecasts are compared with the observed outcomes in order to support organizational learning. Cloud computing methods are used for demanding computational tasks. In cloud computing, clients do not own the computer hardware in question: The services are rented from a third party Internet provider. This procedure reduces capital costs because the health service provider only has to pay for the resources consumed [34].

#### Examples

Comparative assessments can be performed using both databases containing data from historical and current influenza outbreaks as well as from synthetic datasets. For computational efficiency, data and parameter settings from scenario and surveillance modules can be transferred to separate simulation software that runs the comparative analyses [28]. Cloud computing schemes can be used to allocate computationally demanding tasks to computer networks available on the Internet. The analysis software can also be adapted to produce documentation of each step in the evaluation process [31]. Such documentation makes traceable information available for post-processing and quality control.

## Discussion

The PROSPER protocol is to be used for implementation of regional and local information infrastructures supporting response to rapidly emerging infectious diseases. Both policy-makers and public health specialists are exposed to conflicts that arise when trying to create local information systems for pandemic response within centralized health systems. While each of these groups has relied on modern information technology during recent infectious disease outbreaks, insufficient attention has been paid to that the theoretical possibilities of this technology are limited by characteristics of the health system of which the information system is but a part [35]. Managers anticipate improved efficiency and rational allocation of resources, but rational decision-making in pandemics does not automatically emerge from stand-alone or asynchronous decision support systems. While public health specialists seek more effective and equitable response systems, the methodological problems and the expense of many conventional epidemiological approaches continue to limit the usefulness of pandemic surveillance, program monitoring and evaluation. In order to cover and coordinate the key processes in pandemic response, the PROSPER protocol is matched to a generic model for health service delivery and evaluation [36]. For the same reasons, the functions in the infrastructure are adjusted for support of response programs in practice settings, rather than short-term efficacy trials of program components. However, it must be remembered that to build a complete local response system appropriate for the organizational context at hand, the PROSPER protocol has to be complemented with methods for recruitment and coordination of the human resources needed to carry out response actions [37].

Some issues identified in the requirements analysis are not covered by the present version of PROSPER. For example, more research is needed on how pandemic evidence is defined and revised as new infectious diseases progress, and how organizational and intellectual factors influence the uptake of evidence in situations when the timeframe for taking preventive action is short [38]. The implementation of evidence from individual forecasts directly into public health response cycles is not desirable [39], [40]. Therefore, the methods used for synthesizing evidence from predictive modeling will be made explicit in future versions of PROSPER, including guidelines for reporting from different types of modeling. Because of uncertainties associated with even the most advanced current models, their outcomes should be presented as informational resources for pandemic planning, rather than as accurate predictions of intervention or outbreak detection effectiveness [41]. Moreover, the rapid sequence of events during the progress of the 2009 pandemic influenza revealed a functional gap between present methods used for outbreak detection and pandemic forecasting. A technology that could fill this space is nowcasting, i.e. short-term predictions that rely on straight-forward extrapolation of recent observations in time. In meteorology, various nowcasting methods have been developed over the past 20 years for analyses of primary remote sensing data from radar, satellite and lightning [42]. Such nowcasting methods have not yet been included at the functional and technical systems levels in the protocol.

Using PROSPER, basic oversights can be avoided in settings that lack experience of assembling information resources for pandemic response. Interactive modeling packages of ‘what-if analysis’ type, such as FluAid (http://www.cdc.gov/flu/tools/fluaid/) and FluSurge (http://www.cdc.gov/flu/tools/flusurge/) are presently used to inform regional and local policies concerning hospital surge capacity [43] and loss of medical work time [44] when planning pandemic responses. However, use of current ‘what-if’ modeling packages does seldom make it possible to satisfy the conditions for evidence-based reporting according to the STROBE guidelines for observational studies or the SQUIRE guidelines for corresponding reporting from quality improvement studies in health service settings. In evidence-based analysis, users of predictive pandemic modeling should be able to critically inspect all material(s) and model(s) embedded in the analytic resources used. While the need for such transparency has been recognized by the public health modeling community, few models or information structures yet support this feature [45]. The functions included in the PROSPER protocol are defined to be adapted for transparency, e.g. by allowing users to inspect and adjust baseline assumptions used in forecasting. With this transparency, the analytic resources included in the information infrastructure are less likely to be misleading for decision-making at any level.

The PROSPER protocol describes the means required to implement a pandemic information infrastructure regardless of organizational, technical, and financial context. The protocol is preferably applied during inter-pandemic phases, but can also be put into operation by health service providers while an outbreak is progressing through the initial phases, i.e. before it has reached pandemic levels 4 to 6. It can be used to implement support for pandemic response programs not only in environments where sophisticated health information systems are already in place, but also in developing settings with limited access to advanced technology. The protocol allows existing and emerging information technologies to be gradually integrated into the analyses of new infectious diseases, thereby forming an adaptable information infrastructure for synchronized public health response also at national and local levels [46]. It is today mainly applicable to pandemic response, but the protocol can easily be adapted to other human and animal infectious diseases, including bioterrorism [47]. In architecture and urban planning, consensus-based design patterns have been extensively used to transfer design features between different milieus [48]. We have tried to increase the intelligibility of the consensus process in which the protocol was developed by making the goals explicit and providing information on disagreements within the expert group (Text S1) [49].

We have drafted the PROSPER protocol for implementation of information infrastructures that support regional and local pandemic response programs in different organizational settings. To cover key structures and processes in local response, the protocol is based on a generic health service model and its functions are adjusted for community-level analyses of outbreak progress and response program effectiveness. However, if the implementations are to result in reliable and sustainable information infrastructures, corresponding public health theories and practices also have to be integrated. This integration in particular must include the establishment of guidelines for reporting scientific evidence derived from predictive modeling related to infectious diseases.

## Supporting Information

Text S1 **Expert groups involved in collection of requirements data and specification of protocol design.**
(DOC)

Text S2 **Status overview: Surveillance, outbreak detection and predictive modelling.**
(DOCX)

Text S3 **Requirements on data sources and structures.**
(DOCX)

Text S4 **Requirements on functions.**
(DOC)

## References

[1] Krumkamp R, Ahmad A, Kassen A, Hjarnoe L, Syed AM (2009). Evaluation of national pandemic management policies-A hazard
analysis of critical control points approach.. Health Policy.

[2] Dato V, Wagner MM, Fapohunda A (2004). How outbreaks of infectious disease are detected: a review of
surveillance systems and outbreaks.. Public Health Rep.

[3] Ringel JS, Moore M, Zambrano J, Lurie N (2009). Will Routine Annual Influenza Prevention and Control Systems
Serve the United States Well in a Pandemic?. Disaster Med Public Health Prep.

[4] Timpka T, Eriksson H, Gursky E, Nyce J, Morin M (2009). Population-based simulations of influenza pandemics: validity and
significance for public health policy.. Bull World Health Organ.

[5] Lipsitch M, Riley S, Cauchemez S, Ghani AC, Ferguson N (2009). Managing and reducing uncertainty in an emerging influenza
pandemic.. N Eng J Med.

[6] Editorial (2009). Putting influenza A H1N1 in its place.. Lancet Infect Diseases.

[7] Mounier-Jack S, Jas R, Coker R (2007). Progress and shortcomings in European national strategic plans
for pandemic influenza.. Bull World Health Organ.

[8] Petric M, Comanor L, Petti CA (2006). Role of the laboratory in diagnosis of influenza during seasonal
epidemics and potential pandemics.. J Infect Dis.

[9] Jones J, Hunter D (1995). Consensus methods for medical and health services
research.. BMJ.

[10] Rittel H, Webber M (1973). Dilemmas in a general theory of planning.. Policy Sciences.

[11] Ackoff RL (1979). Resurrecting the future of operational research.. J Opl Res Soc.

[12] Buschmann F, Meinier R, Rohnert H, Sommerlad P, Stal M (1996). A System of Patterns: Pattern-Oriented Software Architecture.

[13] von Elm E, Altman DG, Egger M, Pocock SJ, Gotzsche PC (2008). The Strengthening the Reporting of Observational Studies in
Epidemiology (STROBE) statement: guidelines for reporting observational
studies.. J Clinical Epidemiol.

[14] Gennari JH, Musen MA, Fergerson RW, Grosso WE, Crubézy M (2003). The evolution of Protégé: An environment for
knowledge-based systems development.. Int J Hum Comp Stud.

[15] Holm E, Holme K, Mäkilä K, Mattsson-Kauppi M, Mörtvik G (2002). The SVERIGE Spatial Microsimulation Model: Content, Validation,
and Example Applications.. GERUM.

[16] Holm E, Timpka T (2007). A discrete time-space geography for epidemiology: from mixing
groups to pockets of local order in pandemic simulations.. Studies in health technology and informatics.

[17] Sintchenko V, Gallego B (2009). Laboratory-guided detection of disease outbreaks: three
generations of surveillance systems.. Arch Pathol Lab Med.

[18] Smith GJ, Vijaykrishna D, Bahl J, Lycett SJ, Worobey M (2009). Origins and evolutionary genomics of the 2009 swine-origin H1N1
influenza A epidemic.. Nature.

[19] Wahlin K, Grimvall A (2008). Uncertainty in water quality data and its implications for trend
detection: lessons from Swedish environmental data.. Environmental Sci Policy.

[20] Lévy PP, Valleron AJ (2009). Toward unsupervised outbreak detection through visual perception
of new patterns.. BMC Public Health.

[21] Buckeridge DL, Okhmatovskaia A, Tu S, O'Connor M, Nyulas C (2008). Understanding detection performance in public health
surveillance: modeling aberrancy-detection algorithms.. J Am Med Inform Assoc.

[22] Eriksson O (2007). Sensitivity and Uncertainty Analysis Methods: with Applications to a
Road Traffic Emission Model. Doctoral thesis.

[23] Liechti R, Gleizes A, Kuznetsov D, Bougueleret L, Le Mercier P (2010). OpenFluDB, a database for human and animal influenza
virus.. Database (Oxford).

[24] Wirehn AB, Karlsson HM, Carstensen JM (2007). Estimating disease prevalence using a population-based
administrative healthcare database.. Scand J Public Health.

[25] Naditz A (2009). Telenursing: front-line applications of telehealthcare
delivery.. Telemed J E Health.

[26] Ernesäter A, Holmström I, Engström M (2009). Telenurses' experiences of working with computerized
decision support: supporting, inhibiting and quality
improving.. J Adv Nurs.

[27] Davidoff F, Batalden P, Stevens D, Ogrinc G, Mooney S (2008). Publication guidelines for quality improvement in health care:
evolution of the SQUIRE project.. Quality & safety in health care.

[28] Eriksson H, Morin M, Jenvald J, Gursky E, Holm E (2007). Ontology based modeling of pandemic simulation
scenarios.. Studies in health technology and informatics.

[29] Carrat F, Vergu E, Ferguson NM, Lemaitre M, Cauchemez S (2008). Time lines of infection and disease in human influenza: a review
of volunteer challenge studies.. Am J Epidemiol.

[30] Eriksson H, Morin M, Ekberg J, Jenvald J, Timpka T (2009).

[31] Ekberg J, Timpka T, Morin M, Jenvald J, Gursky E, Akan O, Bellavista P, Cao J, Dressler F, Ferrari D, Gerla M (2009). Transparency and documentation in simulations of infectious
disease outbreaks: Towards evidence-based public health decisions and
communications.. Electronic Healthcare.

[32] Hutwagner L, Thompson W, Seeman GM, Treadwell T (2003). The bioterrorism preparedness and response Early Aberration
Reporting System (EARS).. J Urban Health.

[33] Kulldorff M, Heffernan R, Hartman J, Assunção R, Mostashari F (2005). A space-time permutation scan statistic for disease outbreak
detection.. PLoS Med.

[34] Knorr E, Gruman G (2008). What cloud computing really means.. http://www.infoworld.com/d/cloud-computing/what-cloud-computing-really-means-031.

[35] Sandiford P, Annett H, Cibulskis R (1992). What can information systems do for primary health care? An
international perspective.. Soc Sci Med.

[36] Donabedian A (1966). Evaluating the quality of medical care.. Milbank Mem Fund Q.

[37] Perry HN, McDonnell SM, Alemu W, Nsubuga P, Chungong S (2007). Planning an integrated disease surveillance and response system:
a matrix of skills and activities.. BMC Med.

[38] Eccles MP, Armstrong D, Baker R, Cleary K, Davies H (2009). An implementation research agenda.. Implement Sci.

[39] Pappaioanou M, Malison M, Wilkins K, Otto B, Goodman RA (2003). Strengthening capacity in developing countries for evidence-based
public health: the data for decision-making project.. Soc Sci Med.

[40] Armstrong R, Waters E, Roberts H, Oliver S, Popay J (2006). The role and theoretical evolution of knowledge translation and
exchange in public health.. J Public Health.

[41] Halloran ME, Ferguson NM, Eubank S, Longini IM, Cummings DA (2008). Modeling targeted layered containment of an influenza pandemic in
the United States.. Proc Natl Acad Sci.

[42] Wilson J, Crook N, Mueller C, Sun J, Dixon M (1998). Nowcasting thunderstorms: a status report.. Bull Amer Meteor Soc.

[43] Ten Eyck RP (2008). Ability of regional hospitals to meet projected avian flu
pandemic surge capacity requirements.. Prehosp Disaster Med.

[44] Wilson N, Baker M, Crampton P, Mansoor O (2005). The potential impact of the next influenza pandemic on a national
primary care medical workforce.. Hum Resour Health.

[45] Monloney A (2009). Questions raised over response to influenza A
outbreak.. Lancet.

[46] Morse SS (2007). Global infectious disease surveillance and health
intelligence.. Health Aff.

[47] Suk JE, Zmorzynska A, Hunger I, Biederbick W, Sasse J (2011). Dual-use research and technological diffusion: reconsidering the
bioterrorism threat spectrum.. PLoS Pathog.

[48] Alexander C (1979). The timeless way of building.

[49] Raine R, Sanderson C, Black N (2005). Developing clinical guidelines: a challenge to current
methods.. BMJ.

