# Supplementary material for: Requirements and Design of the PROSPER Protocol for Implementation of Information Infrastructures Supporting Pandemic Response: A Nominal Group Study
Source: PLoS One. 2011 Mar 28;6(3):e17941. doi: 10.1371/journal.pone.0017941 (PMC3065450; doi:10.1371/journal.pone.0017941)
Supplement: Text S1 — Expert groups involved in collection of requirements data and specification of protocol design. (DOC) [file pone.0017941.s001.doc]

**Supplementary Information S1. Expert groups involved in collection of requirements data and specification of protocol design. Statements of disagreements with consensus conclusions and comments on literature reviews are listed.**

Collection of requirements data

**Expert panel on data sources (n=8)**

Epidemiology Principal Deputy of Biodefence (US)

Statement of disagreement with conclusions: Expertise on law and political science was missing in the expert panels which may have limited the validity of the conclusions.

Statement regarding interpretation of the literature: None.

Medicine Professor of Social medicine (Swe)

Statement of disagreement with conclusions: None

Statement regarding interpretation of the literature: None.

Statistics Professor of Statistics (Swe)

Statement of disagreement with conclusions: None.

Statement regarding interpretation of the literature: None.

Public health Chief Information Officer at Regional Public Health Agency (Swe)

Statement of disagreement with conclusions: None.

Statement regarding interpretation of the literature: None.

Social and economic geography Professor of Social and Economic Geography (Swe)

Statement of disagreement with conclusions: None.

Statement regarding interpretation of the literature: None.

Medical anthropology Professor of Anthropology (US)

Statement of disagreement with conclusions: None.

Statement regarding interpretation of the literature: None.

Computer science Professor of Computer Science (Swe)

Statement of disagreement with conclusions: None.

Statement regarding interpretation of the literature: None.

Health informatics Associate Professor of Health Informatics (Swe)

Statement of disagreement with conclusions: None.

Statement regarding interpretation of the literature: None.

**Expert panel on analytic functions (n=5)**

Medicine Professor of Social Medicine (Swe)

Statement of disagreement with conclusions: None.

Statement regarding interpretation of the literature: Research on outbreak detection algorithms is reported in epidemiology, statistics, medical statistics, medical informatics, industrial quality engineering, and other related scientific fields. Not all this literature is covered by the PROSPER review citations *per se*, but the main conclusions should be dealt with.

Computer science Professor of Computer Science (Swe)

Statement of disagreement with conclusions: None.

Statement regarding interpretation of the literature: None.

Statistics Professor of Statistics (Swe)

Statement of disagreement with conclusions: None.

Statement regarding interpretation of the literature: None.

Health informatics Associate Professor of Health Informatics (Swe)

Statement of disagreement with conclusions: None.

Statement regarding interpretation of the literature: None.

Cognitive science Consultant on Cognitive Engineering (Swe)

Statement of disagreement with conclusions: None.

Statement regarding interpretation of the literature: None.

Specification of protocol design

**Expert panel**

Epidemiology Principal Deputy of Biodefence (US)

Statement of disagreement with conclusions: Expertise on law and political science was missing in the expert panels which may have limited the validity of the conclusions.

Statement regarding interpretation of the literature: None.

Medicine Professor of Social Medicine (Swe)

Statement of disagreement with conclusions: None.

Statement regarding interpretation of the literature: None.

Statistics Professor of Statistics (Swe)

Statement of disagreement with conclusions: None.

Statement regarding interpretation of the literature: None.

Statistics Assistant Professor of Statistics (Swe)

Statement of disagreement with conclusions: None.

Statement regarding interpretation of the literature: None.

Public health Chief Information Officer at Regional Public Health Agency (Swe)

Statement of disagreement with conclusions: None.

Statement regarding interpretation of the literature: None.

Social and economic geography Professor of Social and Economic Geography (Swe)

Statement of disagreement with conclusions: More as a clarification, there is a paradox inherent in a generic specification of an information infrastructure. A fair amount of implementation detail in a specific setting is required to make the protocol design understandable and convincing. At the same time, a too specific description can make the protocol irrelevant for many other settings.

Statement regarding interpretation of the literature: None.

Social and economic geography Associate Professor of Social and Economic Geography (Swe)

Statement of disagreement with conclusions: Due to space limitations, the presentation of the PROSPER protocol is condensed and cross-referenced. This reduces the readability of the document.

Statement regarding interpretation of the literature: None.

Medical anthropology Professor of Anthropology (US)

Statement of disagreement with conclusions: None.

Statement regarding interpretation of the literature: None.

Computer science Professor of Computer Science (Swe)

Statement of disagreement with conclusions: None.

Statement regarding interpretation of the literature: None.

Health informatics Associate Professor of Health Informatics (Swe)

Statement of disagreement with conclusions: None.

Statement regarding interpretation of the literature:

Cognitive science Consultant on Cognitive Engineering (Swe)

Statement of disagreement with conclusions: Due to space limitations, the presentation of the PROSPER protocol is condensed and cross-referenced. This reduces the readability of the document.

Statement regarding interpretation of the literature: None.
