# Supplementary material for: Requirements and Design of the PROSPER Protocol for Implementation of Information Infrastructures Supporting Pandemic Response: A Nominal Group Study
Source: PLoS One. 2011 Mar 28;6(3):e17941. doi: 10.1371/journal.pone.0017941 (PMC3065450; doi:10.1371/journal.pone.0017941)
Supplement: Text S2 — Status overview: Surveillance, outbreak detection and predictive modelling. (DOCX) [file pone.0017941.s002.docx]

**Supplementary Information S2. Status overview: Surveillance, outbreak detection and predictive modelling**

The WHO’s Global Influenza Surveillance Network (FluNet) collects and processes laboratory data from 94 countries [3]. In 2009 the network consisted of 122 laboratories that collect specimens from patients in their country with influenza-like illness (ILI) . For virus isolation, the laboratories perform preliminary analyses of isolates including virus type and subtype (http://gamapserver.who.int/GlobalAtlas/home.asp). The network provides virologic data that informs trivalent vaccine strain selection twice a year and that is used to rapidly detect influenza A subtypes that may have pandemic potential. National level surveillance based on laboratory data focuses on measuring disease progress to inform prevention policy development, while local level surveillance informs patient treatment and local outbreak response. However, laboratory-based surveillance systems do not have the capacity to follow the progress of local level disease outbreaks, alert health service agencies regarding response, and educate the general public [4]. One main reason for the inadequacy is the low level of microbiologically verified cases. To compensate for this limitation, syndromic surveillance systems have been developed to monitor data from secondary sources, such as national telenursing services [5] or the Internet [6]. Efforts to develop algorithms for early detection of outbreaks based on analysis of data from multiple sources are in progress [7,8].


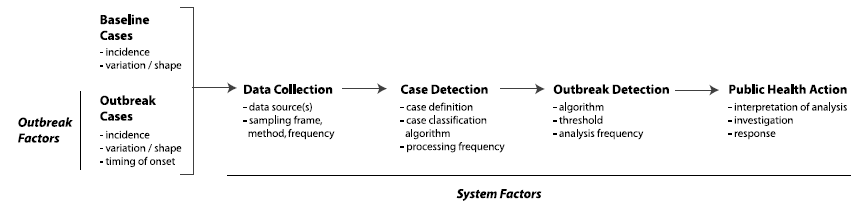


**Figure A1.** Display of associations between outbreak factors and features of outbreak detection systems [9].

In general terms, an outbreak of an infectious disease is easier to detect when the incidence and variation of baseline cases is low relative to the outbreak cases (Figure 1). Outbreaks with a magnitude of less than 10-30 % of baseline have been difficult to detect when operating at high specificity [9]. Also the shape of the outbreak curve influences outbreak detection. Deviations that increase in magnitude quickly over time tend to be detected more rapidly than slowly rising curves. A second set of factors that influence detection are related to the surveillance system. These factors include the data sources, the sampling strategy of the system, the detection algorithm, and the threshold at which an algorithm operates [10]. Systems that monitor a larger proportion of the population at risk are more likely to detect an outbreak. The case detection method, including case definitions and the procedures used to infer whether or not an individual meets case definitions, is therefore important. For instance, outbreaks may fail to be detected if surveillance is only performed at healthcare facilities, because not all symptomatic individuals contact a medical professional. Once cases are identified, a temporal or space–time algorithm is used to analyze the data. Both the historical limits method [11] and the seasonally adjusted cumulative sum (CUSUM) method [12] require five years of baseline data. In comparison, the C1, C2 and C3 family of algorithms used in the Early Aberration Reporting System (EARS) developed by the CDC are closely related to statistical process control methods [13]. These algorithms require less than three years of reference data, and make an alerting decision day by day in a time series by comparing a test statistic to a historical mean calculated from the seven previous days in the baseline data. The performance of these methods has been evaluated with regard to their use in public health surveillance, and it has been found that historical methods do not have a large advantage when compared to non-historical methods [14]. Generally, algorithms that consider multiple days of data at each decision-point tend to outperform algorithms that only consider data from the current day [15].

Beyond surveillance and outbreak detection, computer-based simulations have been used to predict the progress of an outbreak. For instance, containing a pandemic at its hypothesized source through combinations of prophylactic anti-viral medication and social distancing measures has been determined to be feasible [16, 17]. These simulations have required complex models and careful collection of data from multiple sources. However, the validity of these predictions has been weakened by later changes in preconditions, e.g. the development of resistance to second-generation antiviral pharmaceuticals such as Oseltamivir [18]. Similarly, Bayesian estimation using a basic disease model and tracing information from a subset of cases can theoretically allow close to real-time analysis of whether or not an epidemic can be controlled by different interventions [19]. This method assumes that no new cases are introduced to the community, and that all cases are known (asymptomatic persons and underreporting are excluded). Moreover, within the spread of a global pandemic, complex local level processes occur that are seldom modelled in current monitoring and forecasting systems. For example, behavior patterns in populations or groups may rapidly change in a pandemic [20]. This can distort the reliability of low level forecasting [21]. Historical reconstructive simulations have demonstrated that individuals reduce their contact rates in reaction to high levels of mortality during a pandemic [22, 23], and that only the early introduction of social distancing has reduced total mortality [24]. In short, a shortage of real-time outbreak data and a lack of validated methods make pandemic predictions challenging (Figure A2).

**Figure A2.** Outline of the dynamic theory development during emerging infectious disease outbreaks.

**References**

1. Mounier-Jack S, Jas R, Coker R: Progress and shortcomings in European national strategic plans for pandemic influenza. Bulletin of the World Health Organization 2007, 85(12):923-929.

2. Mendel P, Meredith LS, Schoenbaum M, Sherbourne CD, Wells KB: Interventions in organizational and community context: a framework for building evidence on dissemination and implementation in health services research. Administration and policy in mental health 2008, 35(1-2):21-37.

3. Brammer L, Budd A, Cox N: Seasonal and pandemic influenza surveillance considerations for constructing multicomponent systems. Influenza Respir Vir 2009, 3:51-58.

4. Ortiz JR, Sotomayor V, Uez OC, Oliva O, Bettels D, McCarron M, Bresee JS, Mounts AW. Strategy to enhance influenza surveillance worldwide. Emerg Infect Dis. 2009 Aug;15(8):1271-8.

5. Doroshenko A, Cooper D, Smith G, Gerard E, Chinemana F, Verlander N, Nicoll A: Evaluation of syndromic surveillance based on National Health Service Direct derived data--England and Wales. MMWR Morb Mortal Wkly Rep 2005, 54 Suppl:117-122.

6. Ginsberg J, Mohebbi MH, Patel RS, Brammer L, Smolinski MS, Brilliant L: Detecting influenza epidemics using search engine query data. Nature 2009, 457(7232):1012-1014.

7. Buckeridge DL, Burkom H, Campbell M, Hogan WR, Moore AW: Algorithms for rapid outbreak detection: a research synthesis. J Biomed Inform 2005, 38(2):99-113.

8. Buckeridge DL, Okhmatovskaia A, Tu S, O'Connor M, Nyulas C, Musen MA: Understanding detection performance in public health surveillance: modeling aberrancy-detection algorithms. J Am Med Inform Assoc 2008, 15(6):760-769.

9. Buckeridge DL. Outbreak detection through automated surveillance: a review of the determinants of detection. J Biomed Inform. 2007 Aug;40(4):370-9.

10. Bravata DM, McDonald KM, Smith WM, Rydzak C, Szeto H, Buckeridge DL, Haberland C, Owens DK. Systematic review: surveillance systems for early detection of bioterrorism-related diseases. Ann Intern Med. 2004 Jun 1;140(11):910-2.

11. Stroup DF, Williamson GD, Herndon JL, Karon JM. Detection of aberrations in the occurrence of notifiable diseases surveillance data. Stat Med. 1989 Mar;8(3):323-9;

12. Hutwagner LC, Maloney EK, Bean NH, Slutsker L, Martin SM. Using laboratory-based surveillance data for prevention: an algorithm for detecting Salmonella outbreaks. Emerg Infect Dis. 1997 Jul-Sep;3(3):395-400.

13. Montgomery DC. Introduction to statistical quality control. 5th Edition. New York: John Wiley & Sons, 2005.

14. Hutwagner L, Browne T, Seeman GM, Fleischauer AT. Comparing aberration detection methods with simulated data. Emerg Infect Dis. 2005 Feb;11(2):314-6.

15. Izadi M, Buckeridge D, Okhmatovskaia A, Tu SW, O‘Connor MJ, Nyulas C, Musen MA. A Bayesian Network Model for Analysis of Detection Performance in Surveillance Systems. AMIA Annual Symp Proc 2009;Nov 14:276-80.

16. Longini IM, Jr., Nizam A, Xu S, Ungchusak K, Hanshaoworakul W, Cummings DA, Halloran ME: Containing pandemic influenza at the source. Science (New York, NY 2005, 309(5737):1083-1087.

17. Ferguson NM, Cummings DA, Cauchemez S, Fraser C, Riley S, Meeyai A, Iamsirithaworn S, Burke DS: Strategies for containing an emerging influenza pandemic in Southeast Asia. Nature 2005, 437(7056):209-214.

18. Schirmer P, Holodniy M: Oseltamivir for treatment and prophylaxis of influenza infection. Expert opinion on drug safety 2009.

19. Cauchemez S, Valleron AJ, Boelle PY, Flahault A, Ferguson NM: Estimating the impact of school closure on influenza transmission from Sentinel data. Nature 2008, 452(7188):750-754.

20. Seale H, Leask J, Po K, MacIntyre CR: "Will they just pack up and leave?" - attitudes and intended behaviour of hospital health care workers during an influenza pandemic. BMC health services research 2009, 9:30.

21. Ferguson NM: Capturing human behavior. Nature 2007, 446:733.

22. Bootsma MC, Ferguson NM: The effect of public health measures on the 1918 influenza pandemic in U.S. cities. Proceedings of the National Academy of Sciences of the United States of America 2007, 104(18):7588-7593.

23. Caley P, Philp DJ, McCracken K: Quantifying social distancing arising from pandemic influenza. Journal of the Royal Society, Interface / the Royal Society 2008, 5(23):631-639.

24. Hatchett RJ, Mecher CE, Lipsitch M: Public health interventions and epidemic intensity during the 1918 influenza pandemic. Proceedings of the National Academy of Sciences of the United States of America 2007, 104(18):7582-7587.
