# Supplementary material for: Requirements and Design of the PROSPER Protocol for Implementation of Information Infrastructures Supporting Pandemic Response: A Nominal Group Study
Source: PLoS One. 2011 Mar 28;6(3):e17941. doi: 10.1371/journal.pone.0017941 (PMC3065450; doi:10.1371/journal.pone.0017941)
Supplement: Text S3 — Requirements on data sources and structures. (DOCX) [file pone.0017941.s003.docx]

**Supplementary Information S3. Requirements on data sources and structures**

Problem-Requirement A1 **Socio-immuno-geographical representation of communities** To conduct realistic analyses of outbreak detection and intervention effectiveness, the population under risk needs to be represented both with regard to immunity [1], but also in social and behavioral detail [2]. Today, the person-to-person contact patterns in different age, cultural, and social groups are only beginning to become understood [3,4]. A key aspect is that the heterogeneity between individuals and groups with regard to social contacts and disease susceptibility is spatially structured. A substantial part of these spatial differences are compositional in terms of population attributes, e.g. age, education, employment, work income, and social welfare earnings. Other conditions are more directly spatial, such as travel and commuting conditions, and local culture.

Spatial heterogeneity can be regarded as a given structure for disease detection and intervention analyses. This concept refers to a spatially structured relational property of (mixing) groups, not of individuals in isolation. Children and the elderly are examples of subpopulations that may be more susceptible to certain infections as individuals, and therefore when aggregated spatially, increase the disease rates for a specific area. This clustering, however, contributes only a minor part of the spatial heterogeneity relevant for infectious disease transmission and intervention. The major contribution comes from variations in frequency, duration and closeness of personal meetings. The chain of meetings between day care center employee to child, child to parent, parent to work colleague, etc. constitutes the factual route for disease transmission. Therefore, a middle-aged and domestically born business executive speeding between social arenas using several means of public transportation may be the key local agent in infectious disease transmission, despite being less biomedically susceptible than the average person. The configuration of such transmission networks is inherently spatial and spatially heterogeneous. A part of this network heterogeneity can be correlated to the compositional attributes of the local population (to areas where many children, commuters, immigrants or old elderly live), local commuting and labor market conditions, and with local culture and habits, e.g. frequency of meetings in sports clubs and restaurants [5].

These social mixing patterns basically differ substantially between remote, low density regions, the suburbanized countryside, urban ‘township’ districts, the closer suburbs, and core ‘metropolitan’ areas [6]. In the closer suburbs, the mixing is mediated by relatively stable social structures; traditionally employed two-earner families with children living in their own house or flat. Beyond that is the suburbanized countryside, offering inexpensive homes for families with one or less persons employed and with a certain amount of instability, unemployment and turnover. Outside that, the low-density remote rural communities can be found, largely containing the remaining elderly, their employed care-takers, and unemployed closest relatives. The core city, in contrast, mainly contains students and young professionals. They engage intensely in large scale social mixing outside their small flats in the public arenas of the city. Mixing in the city ‘township’ districts is predominately local and ethnical; most of the inhabitants have no reason to travel outside the area. Although the turnover in population varies considerably between these different social and spatial contexts over time, such demographic processes are almost negligible during the time span containing an outbreak of, e.g. a pandemic influenza. A substantial part of the social mixing patterns can thereby be correlated to the composition of the local population and its spatial context [1]. This composition and context can be represented at various levels of detail, and can be preprocessed for later analyses.

Problem-Requirement A2 **Control and visualization of data** **quality and timeliness** Real-time availability of data in terms of quality and timeliness is essential for analyses of outbreak detection effectiveness [1]. The sources of syndromic surveillance data can vary considerably with regard to origin ranging from over-the-counter drug sale records to data on disease-related Internet use. Consequently, the quality of this data can vary to a large extent. Therefore, an information infrastructure must allow for effectiveness analyses to be performed on data sources of differing quality and timeliness, both separately and combined. For instance, acceptable reporting rates need to be compared and analyzed because not all surveillance sites may reach the same reporting rates. In such comparative analyses of quality between time series data, visualization is a powerful support means for the detection of deviations. Completeness of reporting (percent of events or cases recorded in the surveillance system) in such cases is a measure of the representativeness of the data reported.

Problem-Requirement A3 **Explicit representation of population behavior over time** To support gradual implementation of pandemic response, it is necessary to detect outbreaks and to generate updated prognostic information on the actual spread of the infectious agent in the population [1]. However, it is also important to be able to, at any point in time, look for and detect other syndromic transformations. Changes in population behavior may reflect factual spread of the infection as well as precautionary modifications of behavior. Therefore, long-term intervention planning calls for inclusion of other than purely biomedical response measures, e.g. release of situational information from national or international agencies for disease control. For planning of the long-term implementation of pandemic response, interesting questions for analysis thus also include identification of possible changes in the effectiveness of interventions, and novel geographical variations in disease spread caused by changes in population behaviour.

**References**

1. Van Kerkhove MD, Asikainen T, Becker NG, Bjorge S, Desenclos JC, dos Santos T, et al: WHO Informal Network for Mathematical Modelling for Pandemic Influenza H1N1 2009 (Working Group on Data Needs). Studies needed to address public health challenges of the 2009 H1N1 influenza pandemic: insights from modeling. PLoS Med. 2010 Jun 1;7(6):e1000275.
2. Kozlowski LT, Kiviniemi MT, Ram PK. Easier said than done: behavioral conflicts in following social-distancing recommendations for influenza prevention. Public Health Rep. 2010 Nov-Dec;125(6):789-92.
3. Wallinga J, Teunis P, Kretzschmar M. Using data on social contacts to estimate age-specific transmission parameters for respiratory-spread infectious agents. Am J Epidemiol. 2006 Nov 15;164(10):936-44.
4. Mossong J, Hens N, Jit M, Beutels P, Auranen K, Mikolajczyk R, Massari M, Salmaso S, Tomba GS, Wallinga J, Heijne J, Sadkowska-Todys M, Rosinska M, Edmunds WJ. Social contacts and mixing patterns relevant to the spread of infectious diseases. PLoS Med. 2008 Mar 25;5(3):e74.
5. Haugen K, Holm E, Lundevaller E, Westin K: Localised attitudes matter: a study of sickness absence in Sweden. Population, Space and Place 2008, 14(3):189-207.
6. Wenjuan L, Holm E, Lindgren U: Attractive Vicinities. Population, Space and Place 2009,15:1-18.
