# Supplementary material for: Requirements and Design of the PROSPER Protocol for Implementation of Information Infrastructures Supporting Pandemic Response: A Nominal Group Study
Source: PLoS One. 2011 Mar 28;6(3):e17941. doi: 10.1371/journal.pone.0017941 (PMC3065450; doi:10.1371/journal.pone.0017941)
Supplement: Text S4 — Requirements on functions. (DOC) [file pone.0017941.s004.doc]

**Supplementary Information S4. Requirements on functions.**

*Outbreak detection analyses*

Problem-Requirement D1 **Access and adjustments to data** Most outbreak detection systems rely on threshold values derived from baselines calculated from historical data [1]. However, there are several differences between retrospective and real-time outbreak analyses that need to be addressed in response program implementation. One main problem is how to deal with missing data, e.g. reporting delays and censorship. During an ongoing outbreak, data must be analysed as it arrives. Since the purpose is to inform timely interventions, little or no time can be spent on data validation. Preliminary methods to deal with, for instance, case-fatality ratios under such circumstances have been reported [2]. However, data on incubation period and the duration of symptoms are likely to be underestimated during an outbreak and will need to be adjusted later on [3].

Problem-Requirement D2 **Integration of multiple context-specific detection algorithms** In syndromic outbreak detection, no statistical method for sensitivity and uncertainty analysis can be expected to be optimal for all settings. A suitable set of algorithms for real-time examination of outbreak-related data need to be determined for each general outbreak context, i.e. surveillance methods must have been identified that can be applied specifically to outbreaks in communities of similar structure. Statistical outbreak detection methods thus need to be matched to large numbers of outbreak-related conditions or combinations of them. The methods need to handle outbreak detection from such diverse sources as telenursing data, Internet use patterns, and administrative healthcare records [4]. Most present methods for the early detection of disease outbreaks are temporal in nature. These methods are useful for detecting outbreaks that simultaneously affect all parts of the geographical region in question, but may be late in detecting outbreaks that start locally. The scan statistic is one way to solve problems of multiple testing when there are closely overlapping spatial areas and/or time intervals under surveillance [5]. The idea is that there is a scanning window that moves across space and/or time, i.e. the number of observed and expected cases is counted for each location and size of the window. Among these, the most ‘‘unusual’’ excess of observed cases is noted. The statistical significance of this cluster is then evaluated, taking into account the multiple testing that stems from the many potential cluster locations and sizes evaluated. The majority of scan statistics require data that provides information about the geographical and temporal distribution of the underlying population at risk. Such representation of the underlying population is less relevant for syndromic surveillance based on data from, for instance, primary care visits and telenursing calls, since catchment areas and access routes may vary. Recently, however, a prospective space–time permutation scan statistic has been reported that does not require population-at-risk data, and which can be used for the early detection of disease outbreaks when only the number of cases is available [6]. For each location and size, the scan statistic looks at potential one-day as well as multi-day outbreaks in order to quickly detect a rapidly rising outbreak and still have the power to detect a slowly emerging outbreak by combining information from multiple days.

In summary, in situations where many combinations of data sources and other potential outcomes exist and can be surveyed, the algorithms must be combined to minimize false positive alerts and avoid an unnecessary burden on public health professionals. Some combinations of febrile, respiratory and gastro-enteric syndromes may be correlated. When these are clinically relevant and frequent, they should be analyzed as a single entity. The direct approach to dealing with multiple possible symptoms and their combinations is thus to define the occurrence of 'syndromes' as individual entities. Alternately, a more sophisticated approach could use multivariate methods to analyze each individual symptom (data stream) and adjust for the correlation between symptoms.

*Intervention analyses*

Problem-Requirement I1 **Comparative studies of interventions** Updated and optimally validated evidence on expected local effectiveness, rather than ‘on average’ efficacy, of alternative interventions in specific settings [7] is the critical factor in pandemic planning. The effectiveness of pharmaceutical interventions, such as *targeted layered containment* of antiviral treatment and prophylaxis, and non-pharmaceutical interventions, such as quarantine, isolation, school closure, community social distancing, and workplace social distancing needs to be forecasted. Scenarios must allow comparative estimates of intervention effectiveness from indices (Ro, R) of epidemic progress in different societal sectors, strata of working-life, and geographical areas [8]. An alternative methodology for short-term predictions is nowcasting, i.e. forecasting that relies on straight-forward extrapolation of recent observations in time. In meteorology, various nowcasting environments have been developed over the past 20 years for analyses of primary remote sensing data from radar, satellite and lightning (e.g., [9]). Few, if any, nowcasting environments have been reported that can make predictions in an emerging infectious disease area. The model should allow comparisons to be made using this methodology.

Problem-Requirement I2 **Explicit fact and hypothesis management** For comparative analyses to be valid, mechanisms that transfer systematic irregularities into predictive modelling methods need to be identified and retracted. For instance, preliminary hypotheses that rely on assumptions defined for a specific analysis context should not be re-used at the evaluating end of the methods. This would introduce the risk that assumptions that underpin forecasting models become implicitly accepted both in theory construction and in evaluation [10].

**References**

1. Farrington P, Andrews N: Outbreak detection: application to infectious disease surveillance. In: Monitoring the health of populations Edited by Brookmeyer R, Stroup D. Oxford: Oxford University Press; 2004.

2. Ghani AC, Donnelly CA, Cox DR, Griffin JT, Fraser C, Lam TH, Ho LM, Chan WS, Anderson RM, Hedley AJ et al: Methods for estimating the case fatality ratio for a novel, emerging infectious disease. Am J Epidemiol 2005, 162(5):479-486.

3. Brookmeyer R, Blades N: Prevention of inhalational anthrax in the U.S. outbreak. Science 2002, 295(5561):1861.

4. Mostashari F, Hartman J. Syndromic surveillance: a local perspective. J Urban Health 2003;80:i1-i7.

5. Naus J (1965) The distribution of the size of maximum cluster of points on the line. J Am Stat Assoc 60: 532–538.

6. Kulldorff M, Heffernan R, Hartman J, Assunção R, Mostashari F. A space-time permutation scan statistic for disease outbreak detection. PLoS Med. 2005;2(3):e59.

7. Conway PH, Clancy C: Comparative-Effectiveness Research -- Implications of the Federal Coordinating Council's Report. N Engl J Med 2009.

8. Halloran ME, Ferguson NM, Eubank S, Longini IM, Jr., Cummings DA, Lewis B, Xu S, Fraser C, Vullikanti A, Germann TC et al: Modeling targeted layered containment of an influenza pandemic in the United States. Proceedings of the National Academy of Sciences of the United States of America 2008.

9. Wilson J, Crook N, Mueller C, Sun J, Dixon M: Nowcasting thunderstorms: a status report. Bull Amer Meteor Soc 1998, 79:2079-2099.

10. Editorial: Pre-empting a pandemic - fact or fiction? Lancet 2009; May 9;373(9675):1578.
